# Measurement of U and Pu isotopic ratio in environmental samples through MC-ICP-MS integrated to an automated microfluidic column

**DOI:** 10.1007/s10967-025-10097-z

**Published:** 2025-04-24

**Authors:** Shuang Yu Han, Shaun Hemming, Bernard J. Treves Brown, Matthew Alan Higginson, Philip Kaye, Clint A. Sharrad, Scott L. Heath

**Affiliations:** 1 Department of Chemical Engineering, The University of Manchester, Oxford Road, Manchester, M13 9PL UK; 2 School of Ocean and Earth Science, National Oceanography Centre, Southampton, University of Southampton, European Way, Southampton, SO14 3ZH UK; 3 AWE Nuclear Security Technologies, Aldermaston, RG7 4PR UK; 4 Dalton Nuclear Institute, The University of Manchester, Oxford Road, Manchester, M13 9PL UK; 5 Department of Mechanical, Aerospace and Civil Engineering, The University of Manchester, Oxford Road, Manchester, M13 9PL UK

**Keywords:** Microfluidics, Automated separations, Automated analysis, Actinide analysis, Environmental radiochemistry

## Abstract

Accurate identification of isotopes and elements of uranium (U) and plutonium (Pu) in environmental samples from the nuclear fuel cycle is essential for environmental monitoring and safeguarding. A novel microfluidic system coupled with a multi-collecting inductively coupled plasma mass spectrometer (MC-ICP-MS) allows the measurement Pu and U isotope ratios in real time. The application reduces the sample volume required to less than 80 µL with required quantities of analytes being reduced from the conventional micrograms to less than nanograms. This reduction in sample and reagent volume makes the analytical process less resource-intensive and generates less waste; a significant benefit for safer and more efficient analysis operations.

## Introduction

Monitoring anthropogenic sources of actinides in the environment is of particular interest to regulators [1]. This is crucial for timely radiation protection and remediation, reducing hazards, and providing valuable information about the radiological impact on the local biome [2]. Understanding anthropogenic actinides, primarily uranium and plutonium abundance and isotopic composition, also enables relevant agencies to trace their origin, intended use and detect undeclared nuclear activities [3]. Plutonium is an anthropogenic element compared to uranium, which is naturally present in the environment. Pu presence in the environment is mainly attributed to atmospheric atomic weapon testing, which ended in 1975 [4]. Since then, environmental emission of radionuclides is primarily due to nuclear fuel cycle operations and accidents at facilities such as Sellafield, Kyshtym, and Chernobyl [5–7].

The current medium plutonium concentration resulting from weapon testing in the northern hemisphere soil is approximately 10^–13^ g g^−1^ [1, 6]. Accurate analysis of long-lived plutonium isotopes such as ^239^Pu and ^240^Pu, along with short-lived isotopes like ^238^Pu and ^241^Pu provides valuable information about its source [8, 9]. The average global atmospheric fallout has a ^240^Pu/^239^Pu ratio of 0.18, while weapons-grade plutonium typically has a much lower ^240^Pu/^239^Pu ratio of less than 0.08. The signature from nuclear fuel cycle accidents often shows a higher ratio, above 0.30. Additionally, uranium ratios such as ^236^U/^238^U, which vary depending on fuel, burn-up, and reactor type, can also be used in conjunction to determine its regional source and origin [3, 4].

Mass spectrometry, with high instrumental sensitivity and the ability to distinguish between ^240^ and ^239^Pu, became ideal for analysing trace levels of long-lived environmental radionuclides [10–13]. Accelerator mass spectrometry (AMS) and thermal ionisation mass spectrometry (TIMS) are considered the gold standard techniques for the measurement of uranium and plutonium isotopic ratio in the environment [14–16]. Early work on TIMS analysis of nanograms of plutonium loading was sufficient for accurate isotopic analysis with a 0.3% reproducibility within 0.2% of the expected values [17, 18].

However, the presence of natural or anthropogenic ^238^U and the formation of isobaric interferences from uranium hydrides such as ^238^UH^+^ and ^238^UH_2_^+^ cannot be resolved from ^238^Pu, ^239^Pu and ^240^Pu. This requires offline chemical purification of plutonium using extraction or ion exchange resins before mass spectrometry [2, 19]. While current purification protocols are highly effective, they produce solid resin waste, tens of millilitres of potentially radioactive liquid waste that require specialist treatment before disposal, increasing risks of sample contamination from reagents and lab background [20, 21].

TIMS analysis also requires the sample deposition onto a thin filament, a highly-skilled, time-consuming process that requires significant sample interaction and preparation [22]. An alternative mass spectrometry technique for high–precision isotopic measurement is multi collecting-inductively coupled plasma mass spectrometer (MC-ICP-MS) [23]. MC-ICP-MS eliminates errors in sequential scanning in conventional ICP-MS by employing multiple ion counters [24]. The lack of technically challenging filament preparation leads to reduced sample preparation complexity and, potentially, higher sample throughput than TIMS.

MC-ICP-MS requires preparative chemistry to remove isobaric interferences. There is a growing demand to reduce sample and waste volumes needed and generated during analytical separations [25]. Therefore, the increased sensitivity and small sample quantities required by MC-ICP-MS may allow microfluidic inline separations to resolve isobars. Automating the necessary separation stages also reduces potential sources of cross-contamination from manual handling. The combination of flow-injection analysis and direct micro-extraction of plutonium from an environmental swipe has been utilised with mass spectrometry for the separation and online analysis of plutonium and uranium [19]. Integration of microfluidic systems with MC-ICP-MS for radiochemical analysis is currently limited to electrophoretic techniques [22, 26]. Depending on the elements present, carrier solutions must be tailored to specific sample compositions, which is challenging due to the variety of sample compositions across the nuclear fuel cycle.

Automated microfluidic interfaced mass spectrometer systems offer the potential for reducing analytical time and waste. Microfluidic solid phase extraction has been demonstrated for effective radiochemical separations of U and Pu but rarely employed in radiochemical laboratories [27]. The known separation performances of extraction and ion exchange resins offer consistency and can handle a wide range of sample compositions across the nuclear fuel cycle [13, 25, 28, 29].

In this work, MC-ICP-MS integrated single-column microfluidic separation methodologies for Am, U and Pu using extraction and ion exchange chromatography resins UTEVA® and AG® 1 – X8 were developed and validated. The procedure consisted of the separation of Am, Pu and U with a combination of hydrochloric and nitric acids at different molarities, taking advantage of resin affinity for Pu and U at various concentrations. The separation output was interfaced with an MC-ICP-MS to provide qualitative, quantitative and isotopic information regarding the Pu, U and Am-certified reference material (CRMs) used for method validation. The mass bias of the instruments is validated with the use of CRM 145 uranium isotope reference materials. Additional method validation was carried out by directly measuring salt marsh soil samples originating near Sellafield nuclear sites as representative nuclear fuel cycle material. The results of the developed methodology were compared to those obtained from conventional radiometric techniques.

## Experimental materials and methods

### Materials and reference materials

Clear Perspex® (polymethyl acrylate) was purchased from SimplyPlastics (Colchester, UK). Stainless steel frits were purchased from VICI Jour (Schenkon, CH). UTEVA® resins (100 –150 µm) were purchased from TrisKem International (Bruz, FR), AG® 1–X8 anion exchange resins (analytical grade, 100–200 mesh size) were purchased from Bio-Rad Laboratories (Hercules, CA, USA). Optima grade hydrochloric acid (HCl) and nitric acid (HNO_3_) were purchased from Thermo Fisher Scientific (Altrincham, UK).

The simulated environmental test solution for method development contains U, Am and Pu.Prepared from U stocks from the University of Southampton, mixed with Am certified reference material (CRM) PTB 2021–1292, provided by Physikalisch-Technische Bundesanstalt—PTB (Braunschweig, GE). The Pu was provided using CRM—NBL 122, obtained from the New Brunswick Laboratory (Argonne, IL, USA). A separate solution consisting of U CRM material CRM 145 from the New Brunswick Laboratory is mixed with NBL 122 to create a test solution for mass bias correction through sample bracketing, where it is measured before and after the sample to provide an assessment of the mass bias. The sample compositions can be found in Table 1. The bracketing solutions comprised 2 parts per billion of U CRM145 and a diluted Pu CRM NBL 122 solution. The activity of each Pu isotopes in the bracketing solution is listed in Table 2.

**Table 1:** Activity levels of simulated samples used for UTEVA® resin-based separation methodology development. Quantities injected are calculated based on 20 µL injection per analysis

Quantity per injection (ng)	Sample 1 (56 ± 0.1 Bq g^−1^)	Sample 2 (31 ± 0.1 Bq g^−1^)	Sample 3 (2.3 ± 0.1 Bq g^−1^)	Sample 4 (6.3 ± 0.1 Bq g^−1^)
^241^Pu	7.2 × 10^−5^ ± 1.8 × 10^−7^	4.5 × 10^−5^ ± 4.7 × 10^−7^	3.0 × 10^−6^ ± 1.5 × 10^−8^	6.0 × 10^−6^ ± 3.3 × 10^−8^
^240^Pu	6.0 × 10^−3^ ± 2.6 × 10^−5^	3.7 × 10^−3^ ± 1.4 × 10^−5^	2.4 × 10^−4^ ± 1.5 × 10^−6^	5.1 × 10^−4^ ± 3.5 × 10^−6^
^239^Pu	4.6 × 10^−2^ ± 1.2 × 10^−4^	2.8 × 10^−2^ ± 4.5 × 10^−4^	1.8 × 10^−3^ ± 1.0 × 10^−5^	4.7 × 10^−3^ ± 2.4 × 10^−7^
^238^Pu	2.0 × 10^−5^ ± 1.5 × 10^−7^	1.2 × 10^−5^ ± 1.0 × 10^−7^	8.0 × 10^−7^ ± 7.2 × 10^−8^	1.7 × 10^−6^ ± 1.5 × 10^−7^
^241^Am	6.5 × 10^−3^ ± 1.7 × 10^−5^	3.3 × 10^−3^ ± 4.7 × 10^−5^	2.6 × 10^−4^ ± 1.4 × 10^−6^	5.3 × 10^−4^ ± 3.5 × 10^−6^

**Table 2:** Pu activity compositions in the bracketing solution

Bracketing solution	Activity (Bq g^−1^)
^241^Pu	6.0 ± 0.3
^240^Pu	1.1 ± 0.1
^239^Pu	2.3 ± 0.1
^238^Pu	0.3 ± 0.1

The soil samples were collected from salt marshes near the village of Ravenglass (Cumbria, UK). A sediment core was sliced into 1 cm thick samples based on its depth, perpendicular to its ageing direction. Sediment samples at 29–30 cm and 30–31 cm were depth utilised for this study. Soil samples weighing 1.23 and 1.78 g were freeze-dried and then crushed into a powder. The freeze-dried powder was subjected to borate fusion to form glass sample disks, which were later dissolved in concentrated HNO_3_ on a hot plate. The borate was precipitated and removed using a filter column, resulting in a final sample mass of 104 and 112 g of digest in 8 M HNO_3_. The prepared soil sample solution was then stored in a cleaned PFA bottle, from which aliquots were analysed.

### Microsystem design

**Fig. 1:**
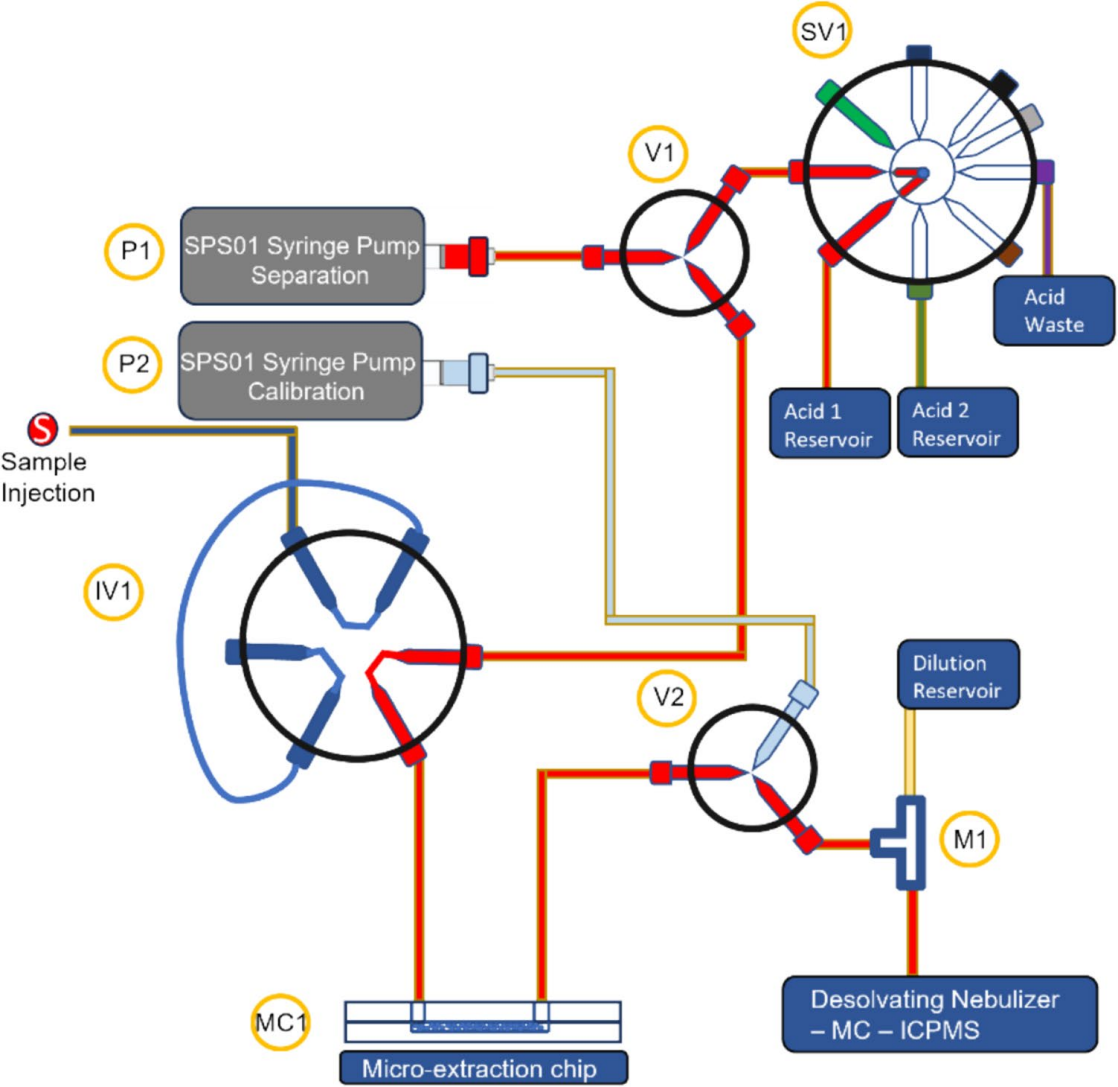
Simplified schematic diagram of the automated separation and isotopic analysis system integrated with MC–ICPMS. P1 = Separation flow pump, P2 = calibration injection pump, SV1 = selector valve for separation flow supply, IV1 = injection valve with sample loop, M1 = mixer, MC1 = packed microcolumn, V1 and V2 = three-way flow control valves, colour diagram throughout this work

Figure 1 shows a simplified schematic of a microfluidic separation-MC-ICP-MS setup. The microfluidic radiochemical separation component of the integrated systems was mounted onto a printed circuit board (PCB) that is approximately 18.5 × 13.3 cm^2^. Pumps and valves are mounted onto the PCB, providing power and interfaces to a PC. The overall arrangement is adapted from previous work on coupling microfluidic separation with conventional ICP-MS.

The system has been modified to reduce its footprint by removing one pump. The elution solution is delivered using a single pump connected to an 8-way supply valve, which allows for the delivery of up to 7 different solutions with just one pump. The microfluidic chips used in this system are described in previous work, and they consist of triple-layer microfluidic chips filled with UTEVA® or AG® 1–X8 resins. The chip interface is achieved with a standardised ¼-28 unified specification receiver port. Additionally, the sample injection volume is controlled by an injection valve equipped with a 20 µL-capacity sample loop made in-house with FEP tubing. The chip designs for the microcolumns were adapted from previous work [30].

### ICP-MS coupling

**Fig. 2:**
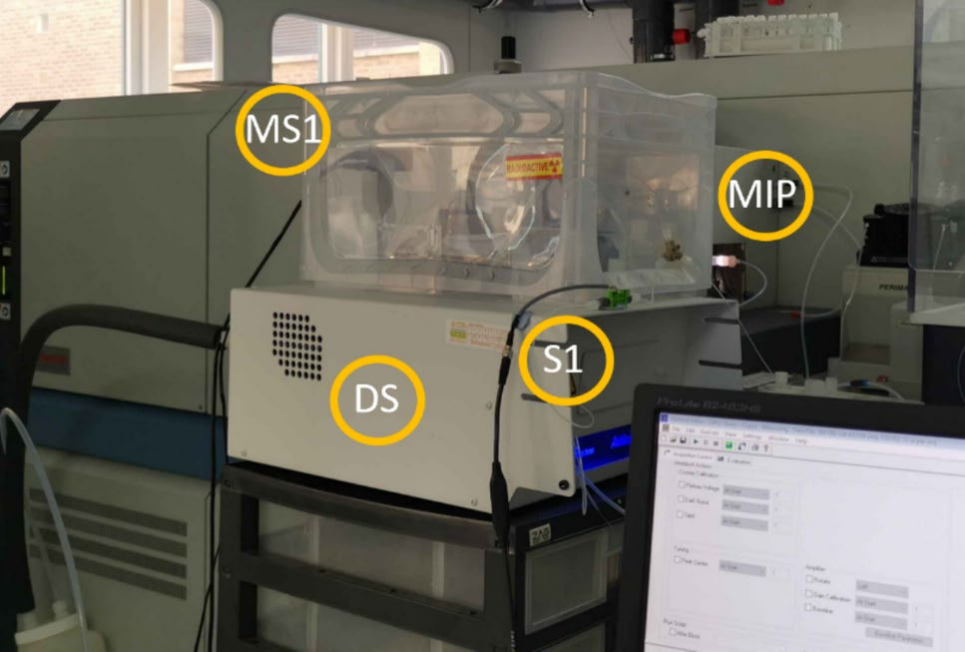
Microfluidic separation platform integrated with an MC-ICP-MS. The components are mounted on 18.5 × 13.3 cm^2^ PCB. MS1 = microfluidic components, mounted within an acrylic containment box, S1 = flowrate sensor, MIP = Thermo Scientific™ Neptune™ MC–ICPMS, DS = Aridus2™ desolvating nebuliser system, Colour diagram throughout this work

The nebuliser system used in this work is a Teledyne Aridus2™ desolvating nebuliser (Thousand Oaks, CA, US) operating at 110 °C, with an argon sweep gas flowrate of 5.3 L min^−1^ and a nitrogen addition gas flowrate of 5 mL min^−1^, connected with the microsystem as shown in Fig. 2

The output of the extraction chromatography section was coupled with dilution flow via a Y–junction connector. The combined HNO_3_ dilution and sample streams were introduced to the nebuliser via self-aspiration, maintaining an average flow rate of 110 µL min^−1^ during sample introduction. The additional dilution flow ensures sufficient quantities of mixed samples reach the nebuliser to achieve the required volumes and to protect the instruments by reducing acid concentration entering the system to below 0.7 M. Long-term exposure to concentrated acid will cause damage to the internal components of MC-ICP-MS and desolvating nebuliser membranes, reducing result reliability. Sensirion (Stäfa, CH) SLI-1000 flow rate sensors monitored the liquid flow rates.

Figure 2 is a photograph of the automated separation integrated with an MC-ICP-MS using a desolvating nebuliser system. The supplementary materials provide a complete list of the parts used.

### Separation methodologies

Depending on the acid and acid concentration, several separation methodologies were developed for the two resins used in this work. The separation stages were implemented with pre-programmed scripts in uProcess™ software.

The overall steps for all the separation methods used for the two resins were identical. The resin columns were first conditioned with the acids used in the first retention stage. Then, the injection valve (IV1) was set to the ‘load’ position, and a syringe was used to inject the sample into the sample loop manually. The automated process then resumes, injecting different acids at pre-determined intervals with designated volumes and flow rates for the sequential separation of Am, U and Pu. The flow rate for the entire operation is set at 20 µL min^−1^. The output of the separation device was then connected to a 100 µL min^−1^ dilution flow using a Y-shaped connector. The MC-ICP-MS signals were collected for all the desired masses as a function of time. The specific arrangements for each separation are listed in the table below (Table 3).

**Table 3:** Automated Separation parameters for isotopic ratio analysis with UTEVA® and AG® 1–X8 resins

	UTEVA®	AG® 1–X8
Method	1	2
Flowrate (µL min^−1^)	20	20
Sample loop size (µL)	20	20
Conditioning Stage	3 M HNO_3_ (100 µL)	3 M HNO_3_ (100 µL)
Stage 1	3 M HNO_3_ (300 µL)	3 M HNO_3_ (1100 µL)
Stage 2	3 M HNO_3_ /0.01 M ascorbic acid (300 µL)	4 M HCl (650 µL)
Stage 3	0.1 M HNO_3_ (300 µL)	N/A

### ICP-MS operation

U and Pu isotope ratios were measured with Thermo Scientific™ Neptune Plus™ multi-collecting ICP–MS, at the University of Southampton. Cups were configured to measure at 235, 238, 239, 240, 241 and 242 using 11-Ω amplifiers. The operating parameters are given in Table 4.

**Table 4:** Neptune Plus™ MC-ICP-MS operating parameters for automated analysis

Acquisition Parameter	Setting
Resolution	Low
Interface	Teledyne Aridus2™
Sample introduction flowrate (µL min^−1^)	Nominally 100
Plasma Power (W)	1300
Sample gas flow (L min^−1^)	1
Auxiliary flow (L min^−1^)	0.8
Data acquisition integration time per cycle (s)	1.049

## Results and discussion

### Mass bias correction

The automated system was validated, and the MC-ICP-MS was calibrated using certified U (CRM 145) and Pu isotopic reference standards (NBL 122). The isotopic standards can be introduced using syringe pump P2, where the outflow is connected to valve V2. V2 switches feed to the T-junction mixer M1 from the microfluidic extraction chip MC1 to the reference standards when required.

The simulated environmental samples used for method development were created by mixing Pu isotopic reference material with U and Am stocks and adjusted to the desired acid molarity by dilution with concentrated acid. The Pu isotopic ratio was used as an internal standard for mass bias correction for the separation method development. The U reference material CRM 145 was injected into the instrument regularly using the calibration pump to monitor instrumental drift and overall stability throughout the experimental sessions. Mass bias correction for environmental samples obtained from salt marshes near the village of Ravenglass (Cumbria, UK) was accomplished using a test solution of a CRM 145 and NBL 122 isotopic reference material mixture.

Due to the chromatography nature of the integrated separation system, the purified actinides enter the MC-ICP-MS like high-pressure liquid chromatography. Where data appears on the MC-ICP-MS as separated peaks as a function of time [2, 22]. Therefore, the data must be collected and treated as transient signals. Three data treatment methods have been previously studied for measuring isotopic ratios using transient signals: linear regression slope (LRS), point by point (PbP) using average ratios obtained by selected points in the separation peaks, and peak area integration (PAI) using the designated area below the peak [22, 31–33].

LRS does not require selecting specific data points; all data points in a particular separation stage are used to evaluate the isotopic ratio of two elements. The advantages of including all data points in a separation stage for the assessment of isotopic ratios are to avoid subjective influence on the results through artificial integration limits or data point selection demanded by PAI and PbP [22]. In LRS, all data points in that particular separation stage are considered equal, and any deviations from an ideal linear fit, whether caused by interfering elements or fractionation, can be identified through the coefficient of determination in the regression curve analysis [33].

In this work, two methods are employed to assess isotopic ratios. For PbP, 15 points before and after each elemental peak are selected for evaluation, where up to two minutes of background is subtracted from the baseline of the selected data point. For LRS, all data points collected for each separation stage are used for evaluation [33].

### Development of Am–U–Pu separation

A simulated mixture containing Am, U, and Pu was prepared using certified reference materials NBL 122, PTB 2021 -1292, and U stock from the University of Southampton. Several dilution levels were prepared to investigate the detection limits for recognisable isotopic ratio determination. Due to radiological limitations to the facility, the maximum activities of the prepared sample mixture were limited to below 60 Bq g^−1^.

#### UTEVA® resin-based separation

The resins suitable for the selective separation of U and Pu include TEVA®, AG® 1–X8, and AC® 1–X8. These resins are designed for extracting Pu from U in HCl [34, 35]. However, in this study, the environmental samples are dissolved in HNO_3_, which limits the available resins and separation methodology. Initially, UTEVA® resin was chosen for the extraction of U and Pu in 3–4 M HNO_3_. UTEVA® is effective for the extraction of U and Pu from HNO_3_ [19]. The adsorbed Pu is retained as Pu(IV); usually, dilute ammonium oxalate, ascorbic acid or hydroxylamine—HCl mixture can be used for the elution of Pu from the retained TEVA®/AG® 1–X8 resins by adjustment of the valence state to unretained Pu(III) [36]. However, hydroxylamine can be potentially hazardous due to the risk of explosion by decomposition in the heated internal chambers. Therefore, ascorbic acid was chosen as the initial candidate for the recovery of Pu from UTEVA® resins [37].

The choice of initial separation conditions is based on previous experience with an automated microfluidic separation system integrated with a conventional ICP-MS for U separation. A flow rate of 1 BV min^−1^ is selected for a 20 µL device, injecting 20 µL of sample per analysis. A sequence of 15 BV 3 M HNO_3_ is used to retain Pu and U and extract Am, followed by 15 BV 3 M HNO_3_/0.01 M ascorbic acid mixture to recover retained Pu. The remaining U is recovered with a 15 BV stage of 0.1 M HNO_3_.

Four sample activity levels were assessed at 56, 31, 6.3 and 2.3 Bq g^−1^. The injected quantities of Pu and Am at each activity level are tabulated in Table 1.

20 µL of diluted simulated environmental sample 3 at 2.3 Bq g^−1^ are injected into the sample loop. The operational flow rate for all stages is set to 1 BV per minute for the 20 µL capacity device. A sequence of 15 BV 3 M HNO_3_ is used to retain Pu and U. Under 3 M HNO_3_, UTEVA® has no affinity for Am. It is not retained and was recovered at approximately 12 min during the separation, collected in a band lasting 220 s.

**Fig. 3:**
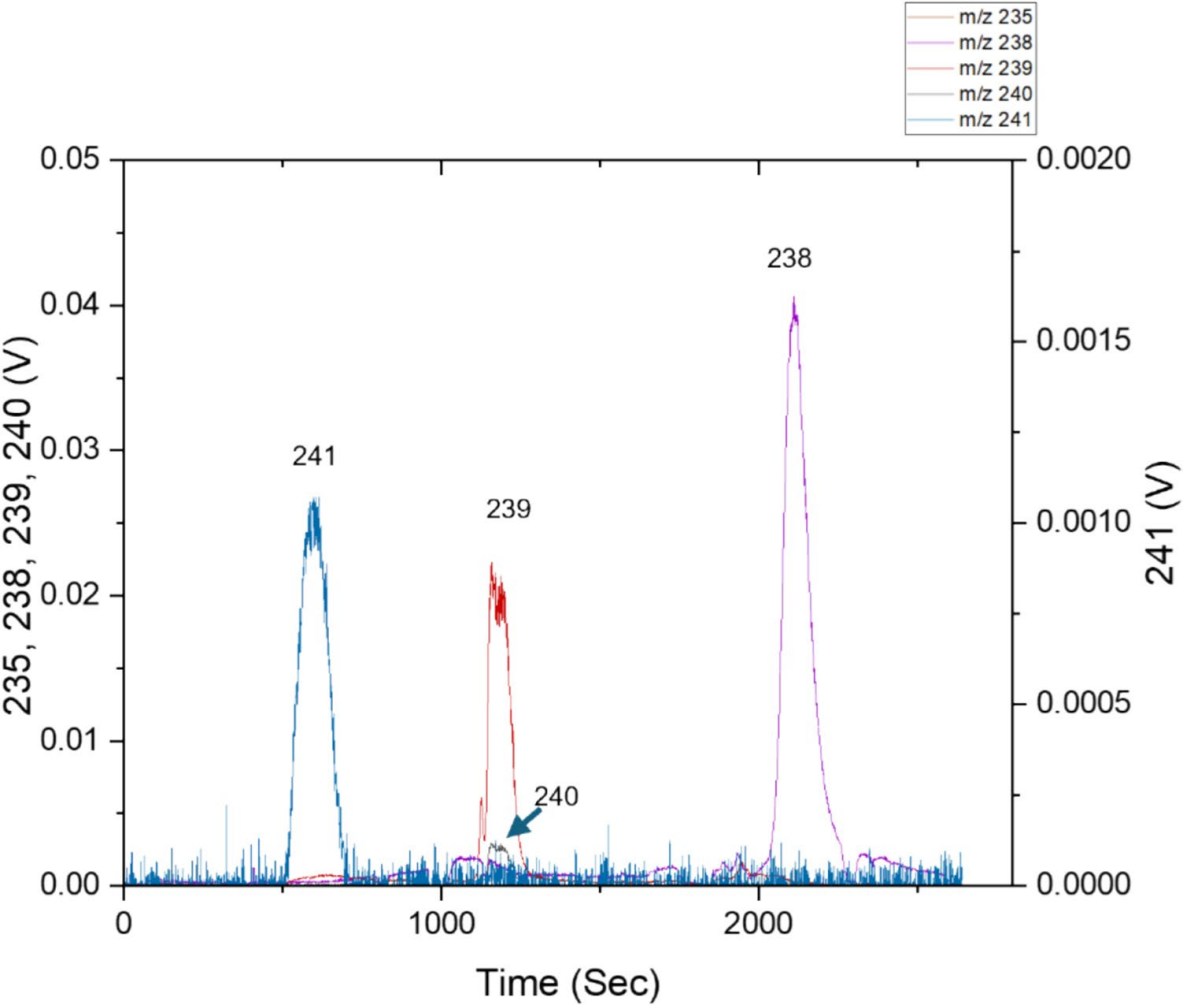
Separation chromatogram obtained after injection of simulated sample 3 (2.3 Bq g^−1^). Three distinct peaks can be identified, with one peak occurring in each separation stage—a significant peak at m/z of 241 during the U/Pu retention stage. Peaks of m/z of 239 and 240 can be detected during the Pu recovery stage. Meanwhile, peaks of m/z of 238 can be detected during the U recovery stage

This recovery band is shown in Fig. 3, occurring at a peak m/z of 241. No m/z = 239 signals were detected within the m/z 241 peak, indicating no Pu breakthrough and interferences from ^241^Pu. Under 3 M HNO_3_, through interaction with extractant diamyl, amylphosphonate (DAAP), Pu and U are retained as Pu(IV) and U(VI) nitrato complexes, driven by the increased nitrate in the system [36, 38]. The retained Pu(IV) is strongly complexed by the resins; decreasing HNO_3_ concentration in the wash stage only has a negligible effect on the overall Pu retention [38].

However, Pu(III) has no interaction with DAAP in the under the presence of nitrate [38]. Therefore, selective elution of Pu can be achieved by reduction of retained Pu(IV) to Pu(III) with an ascorbic acid reductant [37]. Using a combination of HNO_3_ and dilute ascorbic acid, U(VI) remains retained by the resin, while Pu, reduced to Pu(III) by the ascorbic acid, is recovered in a band lasting 230 s. This is demonstrated as peak 2, with two peaks occurring at m/z = 239 and 240. No signals were detectable at m/z of 241; the injected quantities of ^241^Pu were too low for sufficient measurement at 3.0 × 10^−6^ ng. The remaining U was stripped with dilute HNO_3_ and recovered in a band lasting 220 s. Concluding the separation at 44 min.

Using the most dilute sample at an activity of 2.3 Bq g^−1^. ^235^U/^238^U and ^240^Pu/^239^Pu isotopic ratio measurement were possible. The ^241^Am signal, while low, peaked around 0.001 V and was sufficiently distinctive from the background at an Am loading of 2.6 × 10^−4^ ng.

Using PbP, the quantities of ^241^Pu injected were too low to be measured with the current instrument and separation equipment set − up. Significant amounts of m/z 238 signals were measured during Pu recovery using ascorbic acid; around 6% of the total measured m/z 238 signal occurred during the Pu recovery stage.

The quantities injected for ^238^Pu was 8.0 × 10^−7^ ng, which should be too low to be detected by the current MC-ICP-MS setup compared to the higher abundance of ^241^Pu, which was also unobservable. The excessive m/z = 238 signal suggests an incomplete elemental separation between U and Pu, where U is recovered along with Pu during Pu recovery.

**Fig. 4:**
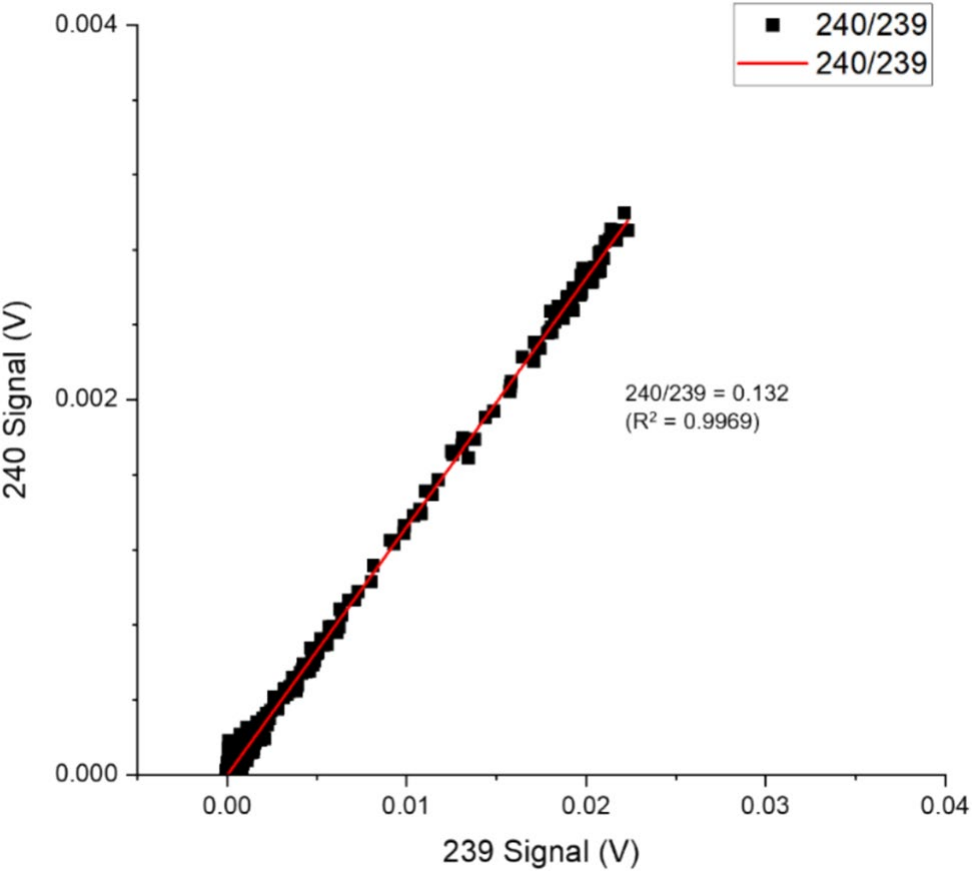
Signal responses from m/z of 240 plotted as a function of 239. The ratio between isotopes was calculated as a function of the gradient of a fitted regression curve to the signal responses, Sample 3

The linear regression curve provides insights into the consistency and reliability of the data by considering all the recorded data during the separation process, as shown in Fig. 4. As part of the regression analysis, the coefficient of determination (R^2^) is also helpful for intuitively evaluating the reliability of the ratio obtained from LRS. The ratios obtained for ^240^Pu/^239^Pu through PbP and LRS were 0.132 ± 0.008 and 0.132 ± 0.012, respectively. These values are within 1.3% (PbP) and 0.9% (LRS) of the decay − adjusted certified reference values for NBL 122. The peak signal monitored for m/z 240 was 0.002 V with 2.4 × 10^−4^ ng of ^240^Pu loaded per analysis.

The Pu isotope ratio measurement reliability increases as the quantities of Pu injected increase. However, more wash volumes and separation time are required to fully recover the injected Pu. Increasing sample activity from 2.3 to 31 Bq g^−1^, increased loading of ^238^Pu by 1500%, doubling the required wash volume and time needed for Pu concentration for the microfluidic separation output to return to baseline. Increasing ^239^Pu and ^240^Pu loading to 3.7 × 10^−3^ and 2.8 × 10^−2^ ng (sample 2) improved the final ratio measured. Compared to the lower activity sample, the ^240^Pu/^239^Pu measured were 0.132 ± 0.005 (PbP) and 0.132 ± 0.004 (LRS).

The relationship between the Pu quantity injected and the measured isotopic data reliability can be more easily seen using ^241^Pu/^239^Pu isotope ratio measured between different samples. At an injected ^241^Pu quantity of 3.0 × 10^−6^ and 6.0 × 10^−6^ ng, the measured LRS isotopic ratio all possess a low coefficient of determination, as the results of low quantities available for measurement. However, while the isotopic ratio provided by LRS for low-level samples is not definitive, the final ratio provided was within 20% of the certified value. Demonstrating the potential effectiveness of the LRS method for environmental samples data analysis to provide a starting point for more sophisticated analysis.

**Fig. 5:**
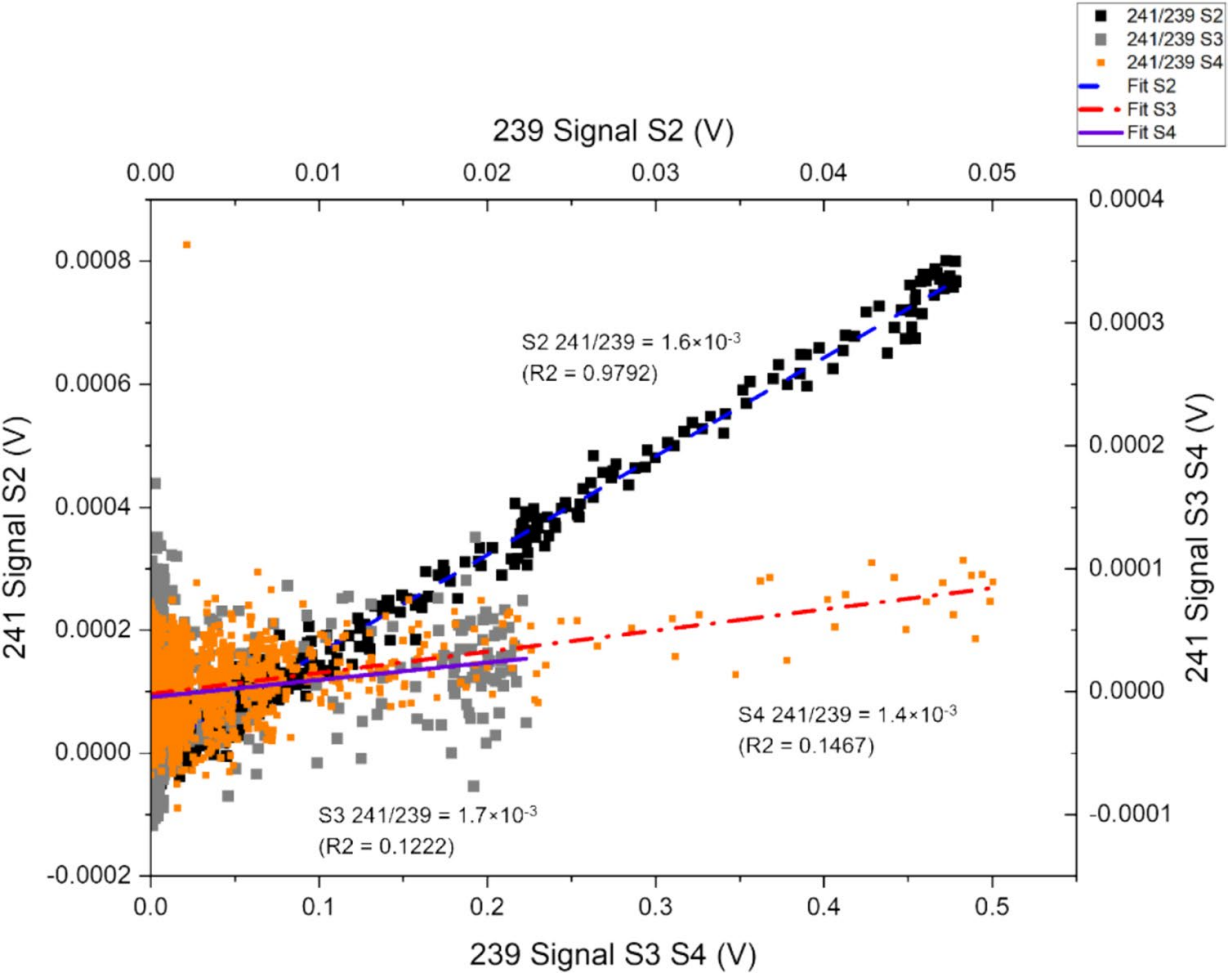
Effect of Increased Pu injection quantities on regression analysis of ^241^Pu/^239^Pu ratio for samples 2, 3 and 4

The coefficient of determination provides an instant insight into the reliability of the isotopic data obtained from LRS analysis. As shown in Fig. 5, for both sample 3 and sample 4, the R^2^ values were below 0.15. The ratio produced by LRS analysis of the highest loading at 4.5 × 10^−5^ ng of ^241^Pu injected (sample 2) was 1.6 × 10^−3^ ± 1.5 × 10^−5^, within 4% of the certified value.

The separation and measurement of m/z 238 was a persistent issue across all separations performed in this work. Compared to ^238^U, the quantities of ^238^Pu were always magnitudes lower, remaining below 0.05 ng per analysis. The presence of ^238^U during the separation process masked the measurement of ^238^Pu, as a small amount of U was leached off from the UTEVA® resins during Pu recovery. Increasing sample concentrations led to an increase of ^238^U and worsened the masking effect.

However, direct injection of diluted ascorbic acid into MC-ICP-MS caused the measured signal intensity to deteriorate throughout several days of experimentation. For example, a 2-ppb sample of CRM 145 used for performance monitoring initially showed a peak measurement of 2.37 V at the beginning of the week. However, by the end of several ascorbic acid experiments, the peak signal measured using the same isotopic reference was reduced to 0.10 V. This is alleviated by increasing the volume of clean-down washes between experiments. However, organic acid eventually causes deterioration and damage to the desolvating nebuliser membrane. This damage reduced signal intensity across all mass-to-charge (m/z) ranges as the samples became trapped within the damaged membrane.

#### AG® 1–X8 resin separation

The current UTEVA®-based separation method, which uses ascorbic acid as a reductant, has been found to cause damage to the nebulization system over both short- and long-term use. The resin choice and separation methodology were revised to exclude organic acids. A new method using AG® 1–X8 resin, which relies only on mineral acids, has been trialled as an alternative. The shift towards using only mineral acids is part of the effort to develop an effective method for automated separation methodologies that can be used directly with mass spectrometry equipment without additional redox or dry-down operations. The activity and concentrations of the Pu samples used for this separation are provided below in Table 5.

**Table 5:** Activity levels of simulated sample 5 used for AG® 1–X8 resin-based separation methodology development. Quantities injected are calculated based on 20 µL injection per analysis

Quantity per injection (ng)	Sample 5 (26 ± 0.04 Bq g^−1^)
^241^Pu	3.3 × 10^−5^ ± 7.5 × 10^−8^
^240^Pu	2.7 × 10^−3^ ± 6.2 × 10^−6^
^239^Pu	2.1 × 10^−2^ ± 4.7 × 10^−5^
^238^Pu	8.9 × 10^−6^ ± 3.1 × 10^−8^
^241^Am	3.0 × 10^−3^ ± 6.9 × 10^−6^

In the AG® 1–X8 separation method, U was no longer retained, and the samples remained in 3 M HNO_3_. However, compared to the UTEVA® based method, the 3 M HNO_3_ wash stage was extended to 30 BV, where Am and U are recovered in this stage. Recovery of Am and U in the same fraction would not affect Am or U measurement as there are no isobaric isotopes between the two elements. Extended wash time would be ideal to remove as much background ^238^U signal as possible before Pu recovery with HCl [34, 35].

**Fig. 6:**
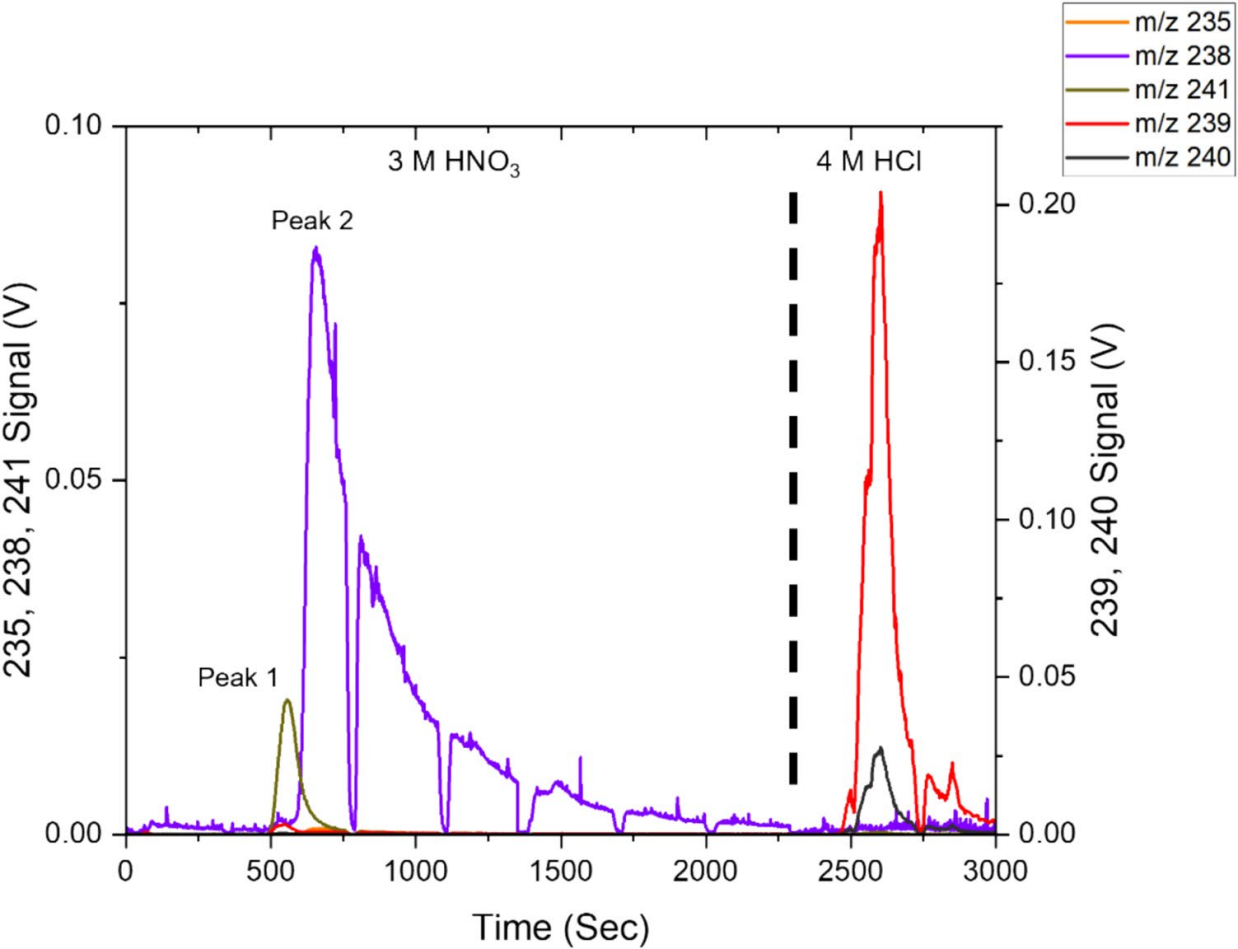
Separation chromatogram obtained after injection of simulated sample 5 (26 Bq g^−1^). Two distinct peaks can be identified. A major peak at m/z of 241 during the Pu retention stage. Peaks of m/z of 239 and 240 can be detected during the Pu recovery stage. Meanwhile, prolonged peaks of m/z of 238 can be detected during the Pu retention stage

However, weak affinity exists between AG® 1–X8 resin and U at 3 M HNO_3_, evident by the extended wash time required to reduce ^238^U to a minimum level of 0.001 V. The extended volume required exceeds the 100 µL capacity of the syringe pump. This results in multiple syringe refills and injections to achieve the 30 BV elution volume requirements. The gaps in solution delivery are evident in Fig. 6 between peak 1 and peak 2 due to pump refilling and restarting of the solution delivery. The interruptions in signal measurements are unavoidable using the automated microfluidic separation device as long as the requirement for elution is above 5 BV. However, this has minimal effect on LRS as reductions in signal intensity are identical and simultaneous for both isotopes.

The persistent interference from ^238^U was effectively reduced compared to UTEVA® based ascorbic acid recovery process. However, there are still difficulties in separating U/Pu; a small but persistent 238 background contribution from U still interferes with the measurement of ^238^Pu despite the extended wash period, as evidenced by the high 238 peak signal measured in the Pu recovery fraction. The measurement of Pu isotopic ratio with 20 µL of a 26 Bq g^−1^ sample using the AG® 1–X8 method is presented in Table 6.

**Table 6:** ^238^Pu/^239^Pu, ^240^Pu/^239^Pu, ^241^Pu/^239^Pu isotope ratios as obtained using LRS and PbP methodology from simulated fuel sample (26 Bq g^−1^), all data used

	^238^Pu/^239^Pu	^240^Pu/^239^Pu	^241^Pu/^239^Pu
Peak signal	238–0.004 V 239–0.204 V	240–0.027 V 239–0.204 V	241–3.5 × 10^−4^ V 239–0.204 V
LRS	1.2 × 10^−3^ ± 1.0 × 10^−3^	1.33 × 10^−1^ ± 1.1 × 10^−4^	1.5 × 10^−3^ ± 5.1 × 10^−5^
Certified	4.32 × 10^−4^ ± 9.1 × 10^−6^	1.31 × 10^−1^ ± 4.6 × 10^−5^	1.5 × 10^−3^ ± 1.8 × 10^−6^

**Fig. 7:**
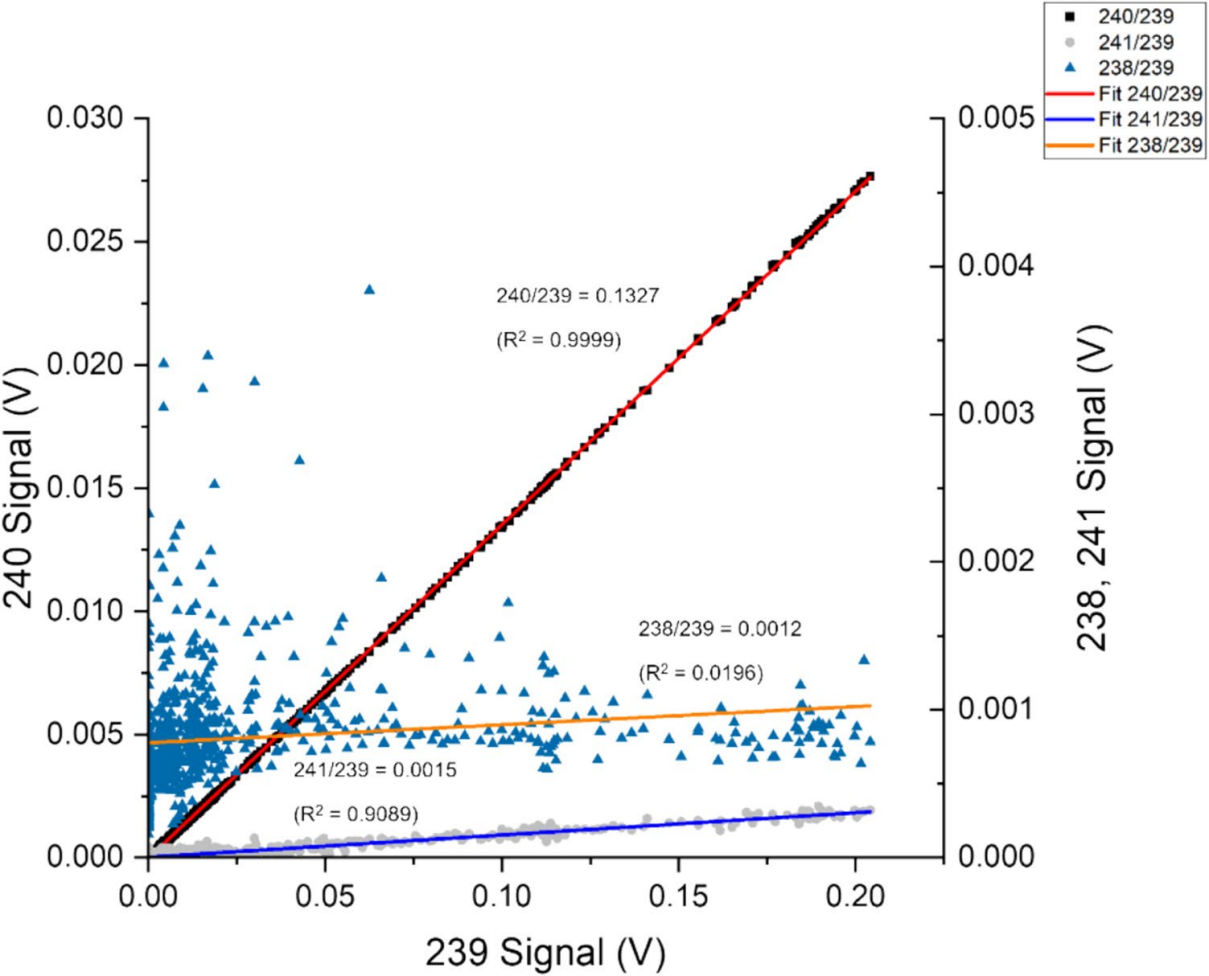
Simultaneous signal responses from m/z of 238, 240 and 241 are plotted as function of 239. The ratio between isotopes were calculated as a function of the fitted regression curve to the signal responses

LRS was modified compared to the original method proposed for high-pressure liquid chromatography (HPLC), where all data points in the particular separation stage were considered. With perfect separation, an increase of 238 signals during Pu recovery should be due to ^238^Pu. However, as U could not be separated entirely, some contribution of the 238 signals during the Pu recovery stage was due to the presence of ^238^U. Specific data ranges need to be rejected below 0.05 V of m/z 239 signals to minimise the ^238^U signal impact on the regression analysis of ^238^Pu/^239^Pu. This impact can be seen in Fig. 7. The cluster of ^238^Pu/^239^Pu at a low 239 signal represents the contribution from ^238^U.

For example, the ^238^Pu/^239^Pu ratio was determined from LRS with unrefined data, considering that the signals across the Pu recovery process were 0.0012. The corrected ^238^Pu/^239^Pu ratio obtained by rejecting low 239 signal ratios were 0.0001. While the data produced are still unreliable, they demonstrate the effective removal of some of the ^238^U influence on Pu ratio measurement.

The small amount of ^238^Pu was still challenging to measure. An extended wash volume of up to 55 (3 M HNO_3_):1 (Sample) was necessary for effective separation at environmental sample quantity at a U/Pu loading ratio of 0.5:1. At the highest ^238^Pu loading of 2.0 × 10^−5^ ng (sample 1), the ^238^Pu/^239^Pu ratio measured via LRS was 6.0 × 10^−4^ ± 6 × 10^−5^ (R^2^ = 0.4979), 39% higher than the certified value. Any quantity below this also produced unreliable ratio measurements. A lower ^238^Pu loading at 1.2 × 10^−5^ ng resulted in a 400% reduction in measured LRS ^238^Pu/^239^Pu ratio to 1.0 × 10^−4^ ± 6.6 × 10^−4^ (R^2^ = 0.01393).

Increasing U quantity would necessitate a further increase in the wash volumes required to minimise m/z 238 contribution from U with the current separation method. Small amounts of ^238^Pu are present in the reference materials used in this work. Due to limited access to ^238^Pu spikes and facilities' limitations on higher activity samples, an additional ^238^Pu could not be spiked for further investigation of the ^238^U impact on ^238^Pu/^239^Pu ratio measurements.

The detection limit of various Pu isotopes using the current separation method and analytical setup is difficult to determine. Traditional detection limit calculations via a calibration/response curve using a known sample are not representative of the contribution due to the chromatographic nature of the separation. However, through this work, under the present instrument configuration using the AG® 1–X8 approach, the sample quantity required for Pu for reasonable isotopic determination can be determined at least within a magnitude. The final Pu quantities required for a reasonable isotopic ratio determination using the automated separations and LRS data treatment are listed below in Table 7.

**Table 7:** Pu quantities required for reasonable (R^2^ > 0.95) isotopic determination using the MC-ICP-MS integrated automated microfluidic separation system and data treatment with LRS

	^238^Pu	^239^Pu	^240^Pu	^241^Pu
Quantities required (pg)	0.1	0.1	0.1	0.1
Concentration for 20 µL sample loop (ng g^−1^)	0.005	0.005	0.005	0.005

As the result of the methodology development, the AG® 1–X8 separation method using HNO_3_/HCl was selected to analyse authentic environmental samples due to the reduced potential for instrumental damage and more effective Pu/U separation.

### U and Pu measurements on environmental samples

The AG® 1–X8 automated separation and analytical methodology was applied for two environmental samples taken from the salt marsh material at 29–30 cm and 30–31 cm within the core. For analysis, 1.2 and 1.8 g of sediment were used to produce 103 and 112 g of digest in 8 M HNO_3_, respectively. 20 µL of the final digest, containing approximately 0.26 and 0.36 mg of the original core samples from each, were introduced into the sample loop. The mass bias was corrected using a mixture of NBL 122 and CRM 145, which were applied using the same analytical methodology before and after the sample separation. Regular CRM 145 runs were carried out using calibration pumps to monitor any instrumental drift or potential acid damage, as observed in ascorbic acid experiments.

**Fig. 8:**
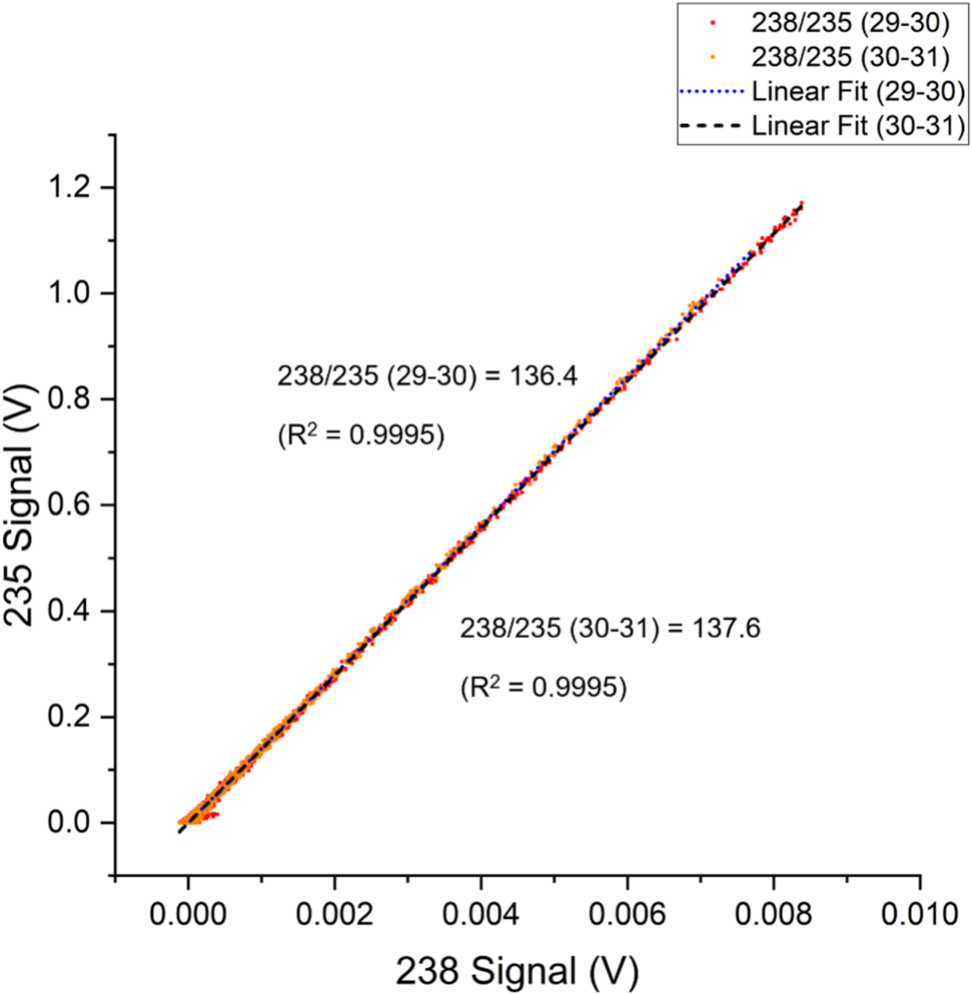
LRS analysis for ^238^U/^235^U for environmental samples taken at 29–30 and 30–31 cm core depths

^238^U/^235^U measured for both core samples were within 1% of the natural ^238^U/^235^U abundance ratio at 136.4 ± 0.4 and 137.6 ± 0.5; the regression data are shown in Fig. 8. With relatively abundant ^235^U, the peak of m/z 235 measured was at 0.007 V during U recovery.

**Fig. 9:**
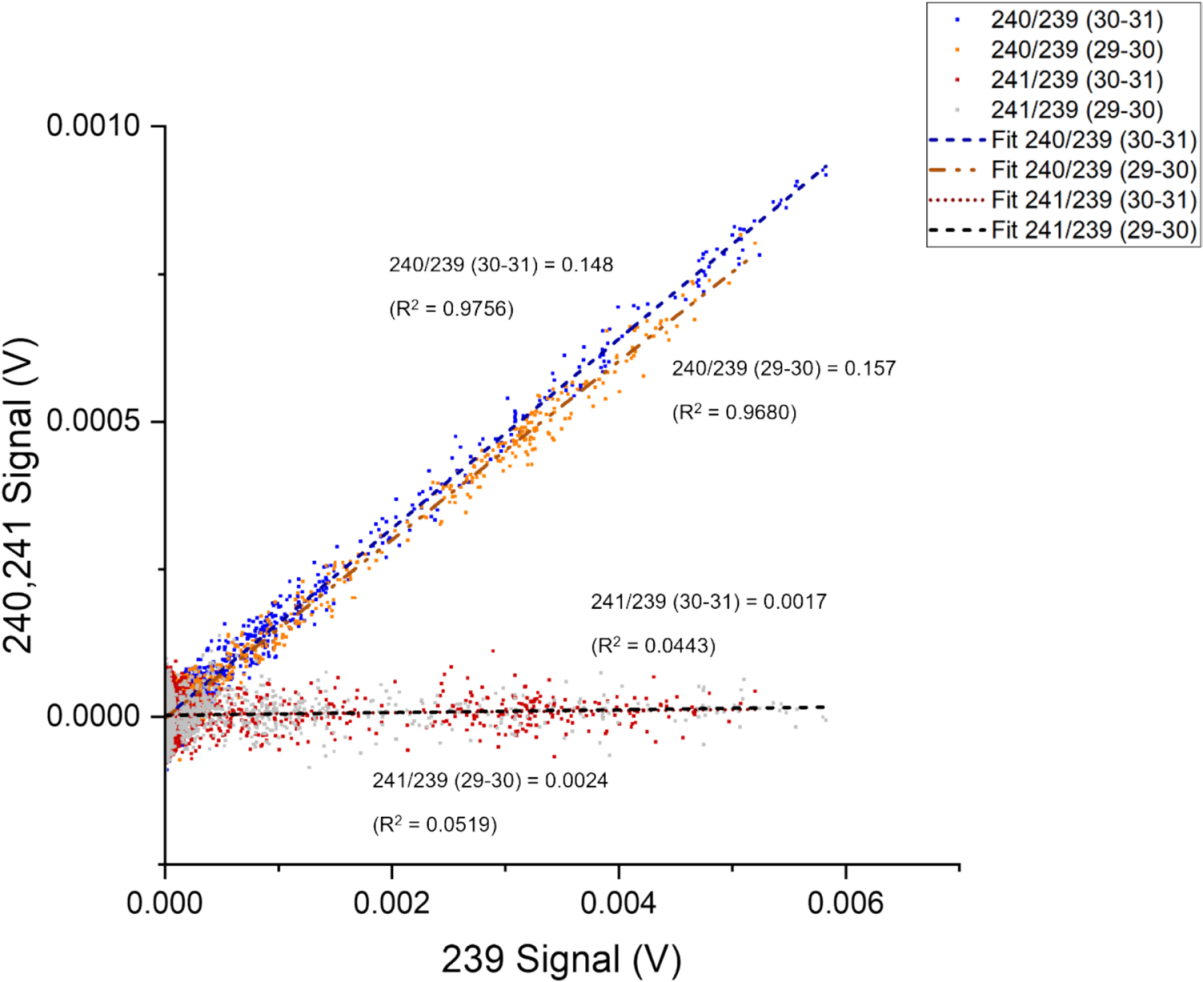
LRS analysis for ^240^Pu/^239^Pu ^241^Pu/^239^Pu for environmental samples taken at core depths of 29–30 and 30–31 cm

As shown in Fig. 9, isotope ratios of ^238^Pu/^239^Pu and ^241^Pu/^239^Pu determined via LRS were unreliable (R^2^ < 0.30). This is likely due to low quantities of ^241^Pu and ^238^Pu injected per analysis. The peak signals for m/z of 241 and 238 measured during Pu recovery were 4.0 × 10^−4^ and 6.6 × 10^−5^ V, respectively. Compared to peak values of simulated samples, ^241^Pu peak below 1 × 10^−4^ V usually indicates insufficient isotopes for effective ratio measurements with the current instrument set-up with the Faraday detectors on the MC-ICP-MS.

As shown in Table 8, ^240^Pu/^239^Pu can be determined with a high coefficient of determination (R^2^ > 0.95) from regression analysis for both core samples. ^240^Pu/^239^Pu = 0.157 ± 0.006 (29–30) and 0.148 ± 0.008 (30–31) after mass bias correction with the available CRM.

**Table 8:** ^238^Pu/^239^Pu, ^240^Pu/^239^Pu, ^241^Pu/^239^Pu isotope ratios were obtained using LRS from assessed environmental core samples

	^238^Pu/^239^Pu	^240^Pu/^239^Pu	^241^Pu/^239^Pu
Peak signal	238–4.04 × 10^−4^ V 239–0.0052 V	240–8.17 × 10^−4^ V	241–6.64 × 10^−5^ V
LRS (29–30)	0.043 ± 0.021 (R^2^ = 0.2333)	0.157 ± 0.006 (R^2^ = 0.9680)	0.002 ± 0.002 (R^2^ = 0.0519)
LRS (30–31)	0.012 ± 0.010 (R^2^ = 0.0376)	0.148 ± 0.008 (R^2^ = 0.9756)	0.002 ± 0.002 (R^2^ = 0.0443)

Quantitative analyses were attempted using certified reference standards as calibration. However, CRM 145 signals used for system performance monitoring were inconsistent during this work. A ± 20% variation in reference material signal intensity was common throughout one day of separation cycles. The resulting variations in signal stability render quantitative analysis with MC-ICP-MS data unreliable. A combined approach was taken to quantify the environmental samples using radiometric and mass spectrometry data.

Pu activities in the remaining core sampling digest were assessed via radiometric methods. ^238^Pu, ^240^Pu + ^239^Pu was assessed via alpha spectrometry after plating 0.04 g of the digest. ^241^Pu were assessed via 4 h count using liquid scintillation. The radiometric measurement of the core sample digest was accomplished by staff at the University of Southampton, and the results are shown in Table 9.

**Table 9:** The activity of Pu isotope in the digest for each core sample was determined via alpha and liquid scintillation spectroscopy

	^238^Pu (Bq g^−1^)	^240^Pu + ^239^Pu (Bq g^−1^)	^241^Pu (Bq g^−1^)
Sample 1 (29–30)	2.50 ± 0.80	11.6 ± 2.0	80.0 ± 42.0
Sample 2 (30–31)	1.70 ± 0.50	13.9 ± 1.9	< LOD

While alpha spectrometry was unable to distinguish between ^239^ and ^240^Pu, the ^239^Pu and ^240^Pu activities can be determined using the isotope ratio provided by the MC-ICP-MS integrated system and the specific activity of each isotope. Activities of ^239^Pu and ^240^Pu in the core samples are ^239^Pu_29-30_ = 7.3 ± 2.3, ^240^Pu_29-30_ = 4.2 ± 0.7 Bq g^−1^ and ^239^Pu_30-31_ = 9.0 ± 4.7, ^240^Pu_30-31_ = 4.9 ± 0.7 Bq g^−1^, respectively.

The quantities of each Pu isotope injected per analysis are presented in Table 10. Compared to the quantities injected to the method limit of detection proposed during method development. ^238^Pu and ^241^Pu are below the required amount of 0.1 pg per cycle, reflected in the uncertainty and the sub-0.1 coefficient of determination in the ^241^Pu/^239^Pu ratio regression analysis. For core samples 29–30, the ^238^Pu/^239^Pu ratio R^2^ value was higher. However, using ^238^Pu and ^239^Pu quantities injected calculated with radiometric and MC-ICP-MS data, the ^238^Pu/^239^Pu ratio is expected to be 1.50 × 10^−5^ ± 0.1 × 10^−5^, hundreds of magnitudes lower than the measured ratio using LRS. The sources of the m/z 238 signal were likely due to the ^238^U contribution.

**Table 10:** Quantities Pu injected, calculated using radiometric data, specific isotope activity, and the ^240^Pu/^239^Pu isotope ratio with MC-ICP-MS

	^238^Pu (pg)	^239^Pu (pg)	^240^Pu (pg)	^241^Pu (pg)
Sample 1 (29–30)	1 × 10^−3^ ± 1 × 10^−3^	0.91 ± 0.16	0.14 ± 0.04	6 × 10^−3^ ± 4 × 10^−3^
Sample 2 (30–31)	1 × 10^−3^ ± 1 × 10^−3^	1.49 ± 0.20	0.22 ± 0.05	< LOD

With the current system setup, radiometric and integrated microfluidic systems are not comparable to TIMS in terms of precision and accuracy. However, compared to up to 20 h required for TIMS, data can be obtained relatively quickly using the integrated microfluidic MC-ICP-MS approach. A significant reduction in sample volumes (20 µL) and Pu quantities required (0.1 pg per isotope per cycle). Through the use of pre-concentration, the automated microfluidic system has the potential for rapid screening of low-activity environmental samples.

## Conclusions

This study aimed to assess the viability and capability of an MC-ICP-MS integrated automated microfluidic separation system for precise isotopic ratio measurement for environmental samples related to the nuclear fuel cycle at picograms of actinides per analytical cycle. The instruments and methodology were designed to be easily adapted for ICP-MS and MC-ICP-MS instruments without further modification, utilising conventional or desolvating nebuliser systems, with the potential for integration in containment.

A methodology for separating Am, Pu and U was developed to measure actinide isotope ratios with the removal of isobaric interferences. Quantities required for reliable Pu isotope ratio determination based on a 20 µL sample loop using the integrated system with simulated environmental samples were investigated and determined to 0.1 pg of isotopes per cycle and at an injection concentration of 0.005 ng g^−1^. Analysis of authentic salt marsh soil samples indicated that linear regression slope (LRS) was an effective data treatment method for analysing chromatograms produced from the integrated system for a representative matrix in the nuclear fuel cycle. For multiple peaks in chromatograms containing isobars, the data range containing the isobars has to be rejected to minimise isobaric effects. This required understanding the separation methodology for selecting the correct stages to analyse desired isotopes. Future applications of non-parametric statistics, such as the Theil–Sen estimator, may aid in data treatment.

However, with the current system arrangement, complete elimination of isobaric interferences was impossible. Neither the integrated system nor the radiometric system was able to achieve the precision of TIMS. The reproducibility of measurements in this work at nano and picogram levels of actinides injected was limited to % instead of ‰ level commonly seen in well-optimised TIMS and MC-ICP-MS measurements. Contamination by ^238^U hinders precise ^238^Pu measurement, both from an imperfect Pu/U separation and U remaining trapped in the fluidic network that was picked up during the proceeding separations. This is alleviated with extended wash stages and increasing actinide quantity injected to above 0.1 pg per isotope per analysis. Lack of access to ^238^Pu spike material due to limitations on the activities of the samples suitable to the MC-ICP-MS instrument facility prevented further investigations of U/Pu separation methodology.

The developments of a mass spectrometry integrated automated microfluidic separation system demonstrated gains in reducing sample volume, actinide quantities, time per analysis required and waste production. Further work is necessary to develop a more effective U/Pu separation methodology, using different resins or solutions for improved actinide separation and recovery. Adapting the existing instrument setup and testing it within a fume hood or glovebox integrated MC-ICP-MS instrument would be of interest. Once developed, it would be of interest to investigate a broader range of materials from the nuclear fuel cycle with different isotopic compositions.
